# Ileal Pouchitis With Endoscopic Pictures

**DOI:** 10.7759/cureus.14778

**Published:** 2021-04-30

**Authors:** Hassam Ali, Abeera Sarfraz, Hadeera Ali

**Affiliations:** 1 Internal Medicine, East Carolina University, Vidant Medical Center, Greenville, USA; 2 Internal Medicine, Federal Medical and Dental College, Islamabad, PAK; 3 Internal Medicine, CMH Institute of Medical Sciences, Bahawalpur, PAK

**Keywords:** pouchitis, ileal pouchitis, ulcerative colitis

## Abstract

Ulcerative colitis (UC) is an inflammatory disorder, and almost one-third of UC patients ultimately undergo surgical interventions because of complications or refractory disease. Current restorative proctocolectomy with ileal pouch-anal anastomosis (IPAA) is the standard intervention for severe chronic UC with refractory disease. Several complications associated with this procedure can occur, including anastomotic leak, sepsis, and pouch ischemia. The most frequent long-term complication is pouchitis, an idiopathic inflammatory condition involving the ileal pouch. Presentations may vary but include stool frequency, urgency, incontinence, fatigue, malaise, and fever, less commonly bloody stools. We report a case of ileal pouchitis in a young patient, two years after proctocolectomy with IPAA responsive to antibiotic treatment. Our case supports that imaging studies like flexible sigmoidoscopy are necessary to rule out other disorders in patients with pouchitis.

## Introduction

Pouchitis is an inflammatory disorder and a common complication of ileal pouch-anal anastomosis (IPAA), in patients with ulcerative colitis (UC) and familial adenomatous polyposis (FAP) [1,2]. The usual presentation includes diarrhea (increased stool frequency) and fecal urgency. Despite an excellent functional outcome after proctocolectomy with IPAA in patients with UC, pouchitis may present in 80% of patients, with or without extraintestinal manifestations, in a lifetime [3,4]. Pouchitis is more prevalent in patients with FAP than UC, although the age at its incidence remains earlier in UC patients [5]. Due to its occurrence in inflammatory diseases, a genetic component is a probable factor in its incidence. Genetic polymorphisms, especially those of interleukin (IL)-1 receptor antagonist, have been linked to increased risk of pouchitis [6].

## Case presentation

**Figure 1:**
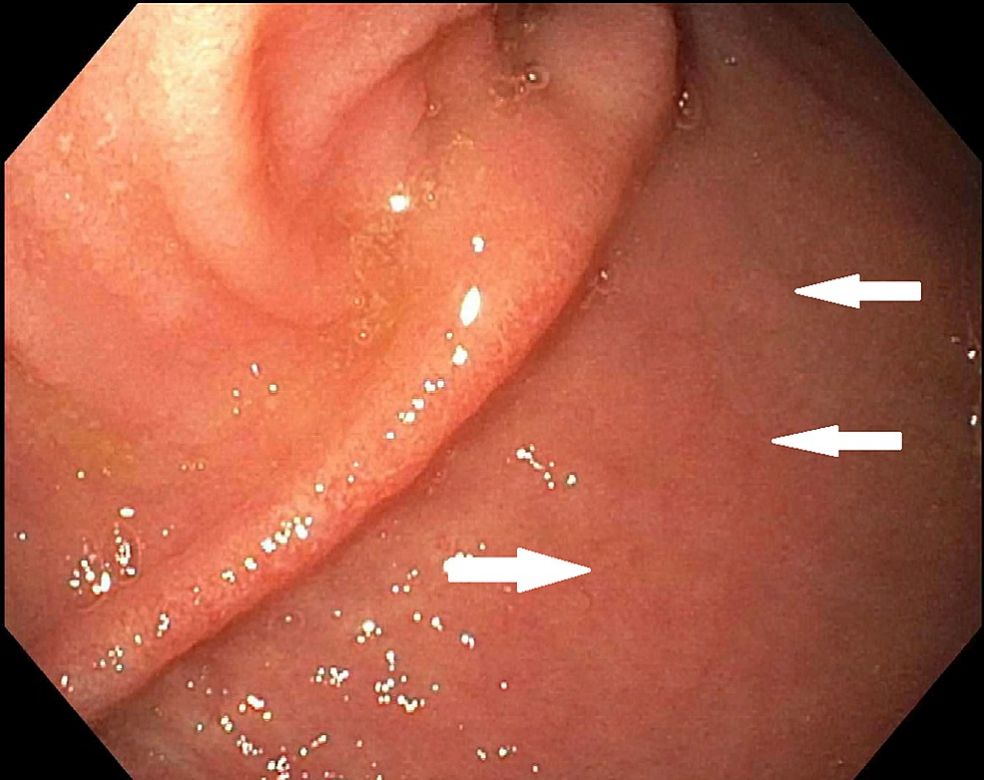
Pouchitis after ileal pouch-anal anastomosis (J-pouch) in ulcerative colitis (white arrows showing erythema).

A 25-year-old female presented with a one-week history of abdominal pain and watery diarrhea. She had a past medical history of papillary thyroid carcinoma with complete thyroidectomy, UC status post colectomy with IPAA (J-pouch). Her pain was most intense in the left lower abdominal quadrant and only relieved by heating pads. She stated an increased frequency of abdominal bowel movements (her regular bowel movements were four/day). She denied any environmental exposures, dietary changes, fevers, chills but stated malaise. The patient was initially placed nil per oral and started on intravenous fluids for dehydration. Her laboratory works up for inflammatory markers, and routine workup was negative, including thyroid panel. Her stool osmolality was 309 mOsm/kg, consistent with secretory diarrhea and abdominopelvic CT scan revealed J-pouch thickening without evidence of bowel obstruction, ascites, adenopathy, or pneumoperitoneum. The Clostridium difficile toxin was negative and stool culture failed to grow anything. Due to her history of UC and surgical intervention, she underwent flexible sigmoidoscopy with biopsy by gastroenterology and was noted to have a patchy area of mild erythematous mucosa (Figure 1). A tentative diagnosis of ileal pouchitis was made due to erythema on sigmoidoscopy.

The patient was started on ciprofloxacin and metronidazole and discharged with a follow-up in the clinic. Her biopsy later revealed severe acute inflammation and degenerative changes without adenomatous dysplasia or malignancy. She received ten days of ciprofloxacin 500 mg twice a day and metronidazole 500 mg twice a day with resolutions of symptoms.

## Discussion

Pouchitis can present with various clinical manifestations, and patients can have an acute or chronic presentation. The spectrum of presentation ranges from increase stool frequency, urgency, abdominal pain, and fecal incontinence. Patients may have associated pelvic or cramping abdominal pain that may not be relieved by a bowel movement. Despite a lack of bloody bowel movements, pouchitis can result in iron deficiency anemia [7,8]. In case of systemic symptoms like fevers, malaise, chills, night sweats, and weight loss, an infectious source of pouchitis should be considered [8].

Ileal pouch endoscopy with biopsy is primarily performed for evaluation in case of a suspected cause of pouchitis. Endoscopically, a clinician may notice erythema (as in our case), erosion or ulceration of mucosa, and increased friability [9]. Typically, additional imaging is not obtained; but to rule out differentials and to assess the presence of mucosal and transmural disease activity, CT scan, pouchography, and MRI can be utilized [10]. Functional abnormalities, for example, pouch manometry, can also be utilized for assessing paradoxical contractions in dyschezia. Pouchitis therapy aims to relieve symptoms and promote mucosal healing. Histologic improvement is emerging as an additional component of disease remission [11]. Treatment response includes improved bowel movement frequency to baseline and better symptom control. Stool culture and susceptibility testing should be done if the patient is not responsive to antibiotics [12]. Typically 14 days of metronidazole 500 mg twice a day or ciprofloxacin 500 mg twice a day can be utilized [12]. No symptomatic recovery despite four weeks of antibiotic therapy can be labeled as antibiotic-refractory pouchitis. Therefore, four weeks of combined ciprofloxacin and metronidazole can be given in refractory cases based on susceptibility results [12,13].

In the case of refractory pouchitis, it may be essential to repeat biopsy to exclude any concurrent infection. Blood tests to rule out primary sclerosing cholangitis and stool specimen for Clostridium difficile should also be obtained. Refractory pouchitis is the leading cause for pouch failure, ending in pouch excision or permanent ileostomy. A chronically inflamed pouch can increase the risk of cellular dysplasia or cancer [14,15]. Due to antibiotic-based therapy, the management of antibiotic-refractory pouchitis remains a challenge. A high dose of probiotics may effectively prevent pouchitis onset, relapses and can help manage chronic pouchitis refractory to antibiotics [16,17]. Anti-inflammatory agents, biologic therapy, and immunomodulators may also be employed [18].

## Conclusions

Pouchitis is the most prevalent long-term unfavorable sequela of IPAA after restorative proctocolectomy. The natural history of pouchitis is poorly understood. Pouchitis has a wide range of clinical presentations, disease course, and the prognosis is often good. The key to appropriately manage pouchitis is an accurate diagnosis and its classification. It is important to consider the cost-effectiveness of diagnostic interventions as well. Treatment of pouchitis is mainly antibiotic-based. Maintenance of remission in antibiotic-dependent pouchitis and management of antibiotic-refractory pouchitis is a challenge. Secondary causes for refractory pouchitis should be excluded. 
